# Minimally Invasive Approach for Diffuse Idiopathic Skeletal Hyperostosis (DISH)-Related Vertebral Fractures: A Case Report on Combining Vertebral Cement Augmentation and Cement-Augmented Pedicle Screw Instrumentation

**DOI:** 10.7759/cureus.49550

**Published:** 2023-11-28

**Authors:** Takaki Inoue, Hiroyuki Motegi

**Affiliations:** 1 Department of Orthopaedic Surgery, Chiba Aoba Municipal Hospital, Chiba, JPN

**Keywords:** vertebral body stenting, cement-augmented pedicle screw, vertebral cement augmentation, vertebral fracture, diffuse idiopathic skeletal hyperostosis

## Abstract

Diffuse idiopathic skeletal hyperostosis (DISH)-related vertebral fractures often require surgical intervention due to associated spinal instability and neurological deficits. This study presents a minimally invasive approach that utilizes vertebral cement augmentation and cement-augmented pedicle screw (PS) instrumentation to manage DISH-related vertebral fractures. We present an 87-year-old male patient with a T11 vertebral fracture associated with DISH. Despite the patient's advanced age and comorbidities, he underwent a successful surgical procedure, achieving relatively short-segment fixation by combining vertebral cement augmentation and cement-augmented PS instrumentation. After the surgery, the patient's lower back pain subsided, facilitating a return to normal activities. Radiographic evaluation at the six-month postoperative stage confirmed the maintenance of vertebral body reduction with no indications of implant failure. In DISH-associated vertebral fractures, the combined application of vertebral cement augmentation and cement-augmented PS instrumentation offers a minimally invasive solution that expedites fracture stabilization and enhances patient outcomes. This approach offers the potential for effective fracture stabilization and a significant reduction in postoperative complications, holding promise for managing challenging cases in this patient population.

## Introduction

Diffuse idiopathic skeletal hyperostosis (DISH), a systemic condition leading to the ossification of ligaments and entheses, is diagnosed when ossification of the anterior longitudinal ligament is present on spine radiographs over at least four consecutive levels [1]. Vertebral fractures associated with DISH present several challenges. Fractures in the fused segments of ankylosed vertebrae tend to form three columns, with a high risk of post-traumatic neuropathy due to instability of the fracture site owing to spinal immobilization and long lever arms [2-5]. Furthermore, the diminished mobility of the vertebral body in DISH may result in decreased bone mineral density due to stress shielding [6]. In addressing DISH-related vertebral fractures and minimizing the risk of implant failure, it was advisable to extend the spinal fusion to encompass at least three vertebrae both above and below the fracture site [2]. On the other hand, DISH-related vertebral fractures often occur in older patients with a high prevalence of comorbidities, with one study reporting a mortality rate as high as 38.1% [2]. Due to the elevated incidence of postoperative complications in DISH patients, minimally invasive and rigid fixation procedures are preferred.

Cement augmentation procedures, including vertebroplasty (VP), balloon kyphoplasty (BKP), vertebral body stenting (VBS), and cement-augmented pedicle screw (PS) instrumentation, have emerged as a tailored and practical solution for achieving fracture stabilization [7-10]. Among vertebral cement augmentation procedures, the VBS procedure has shown a significant decrease in the loss of reduction due to the addition of a metallic stent [11]. We present a case of a DISH-related vertebral fracture that achieved relatively short-segment fixation through a combination of VBS and cement-augmented PS instrumentation.

## Case presentation

**Figure 1:**
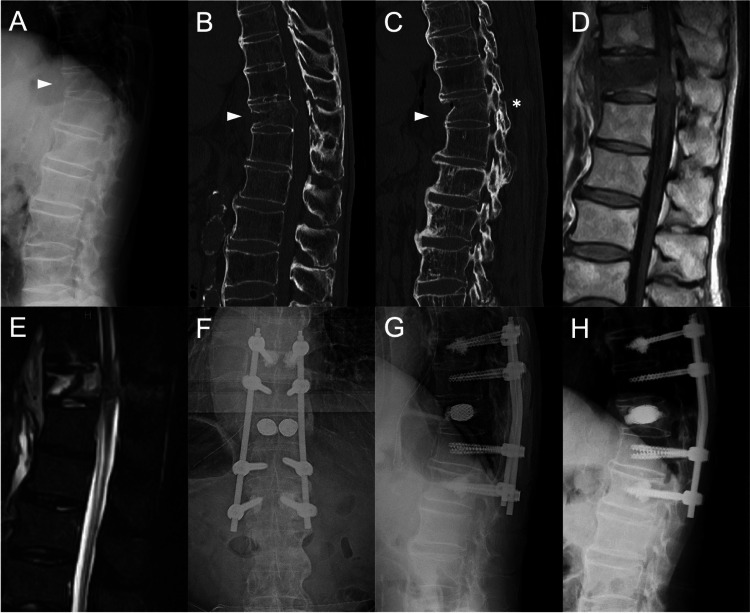
Case A. Preoperative radiographs; B. Median sagittal CT image; C. Paramedian sagittal CT image; D. T1-weighted MRI image; E. STIR MRI image; F. Anterior-posterior radiographs after one week of surgery; G. Lateral neutral radiographs after one week of surgery; H. Lateral neutral radiographs after six-month surgery. CT: computed tomography; STIR: short inversion-time inversion recovery; MRI: magnetic resonance imaging

The case involved an 87-year-old male patient with a history of cerebral infarction and hypertension. He presented with acute lower back pain, as assessed by the visual analog scale (VAS), reaching 9/10, following a fall, necessitating hospitalization. There were no neurological abnormalities. Radiographic evaluations revealed a T11 vertebra fracture (indicated by an arrowhead) and continuous ossification bridges across more than four vertebrae. Initially, the spinal segment cobb angle was kyphotic at 19.0°, with the anterior wall height measuring 15.6 mm (Fig. 1A). Furthermore, a bilateral facet fracture (indicated by an asterisk) was identified in the computed tomography (CT) images (Fig. 1B, 1C). Magnetic resonance imaging (MRI) confirmed the T11 vertebral fracture and associated posterior ligamentous injury (Fig. 1D, 1E). Further analyses, including bone density and biochemical bone markers, collectively confirmed the presence of osteoporosis. Based on this clinical data, the patient was diagnosed with a three-column fracture of T11 (categorized as AO type B2) with DISH. Following hospitalization, the patient persisted in experiencing severe lower back pain, which did not abate even with the use of analgesics and external bracing. Consequently, surgical intervention was performed, encompassing the T9-L1 vertebral levels, which combined VBS and cement-augmented PS instrumentation. The procedure was conducted under general anesthesia and biplanar fluoroscopic guidance, with the patient lying prone. The VBS procedure (VBS; Depuy Synthes, Switzerland) was carried out at the T11 level. Bilateral inflatable balloons with stents were employed to gradually restore the vertebral bodies. Following the removal of the balloon tamp, 6 ml of polymethylmethacrylate (PMMA)-based cement was injected into the T11 vertebra. Subsequently, percutaneous PS fixation was performed, extending to two vertebrae above and below the fracture segments with the insertion of each screw. For the T9 and L1 vertebral bodies, cement-augmented PS systems (VIPER, Depuy Synthes, Switzerland) were used to introduce PMMA-based cement, with 1 ml of cement injected into each screw. The rod was passed under the muscle and firmly attached to the screw head. Following the procedure, the vertebral segmental cobb angle was subsequently reduced to 6.8°, and the height of the anterior wall measured 21.6 mm (Fig. 1F, 1G). Post-surgery, the patient reported relief from his lower back pain. One month after the procedure, the VAS score was reduced to approximately 1 point. The brace was utilized for three months, and subcutaneous denosumab (60 mg) injections were administered every six months. The six-month follow-up revealed the resolution of the lower back pain, with a VAS score of 0/10, enabling the resumption of normal activities without the need for a brace. Radiographic evaluation demonstrated the maintenance of vertebral body reduction, with the vertebral segmental cobb angle measuring 7.3° and the anterior wall height at 20.8 mm (Fig. 1H).

## Discussion

Cement augmentation procedures offer an attractive option for the treatment of vertebral fractures in patients with DISH, providing rapid and effective fracture stabilization. Particularly in vertebral fractures with 3-column injuries associated with DISH, the combined application of VBS and cement-augmented PS instrumentation enables robust fixation with short segments.

Cement augmentation procedures represent a compelling choice for the management of vertebral fractures in DISH patients, as they offer rapid and effective fracture stabilization. Vertebral cement augmentation procedures, including VP, BKP, and VBS, have emerged as valid techniques for anterior column reconstruction in thoracolumbar vertebral fractures [7-9]. These approaches provide minimally invasive methods and rapid pain relief. Biomechanically, burst fractures can significantly decrease spine stiffness by up to 47.5% compared to an intact spine, but the instant stiffness of the spine was recovered to 107.8% of the intact condition by VP [8]. The VBS procedure, introduced as a new option for vertebral cement augmentation, has an advantage over BKP in that the stent maintains the restored vertebral body height after intravertebral reduction [11]. Some reports have demonstrated the usefulness of cement augmentation for vertebral fractures in ankylosing spines, including DISH [12-14]. Tsuchikawa et al. reported improved VAS and Oswestry Disability Index (ODI) scores in patients undergoing BKP surgery for fractures of the distal or distally adjacent vertebrae of DISH [14]. Even without posterior fixation, cement augmentation procedures contribute effectively to stabilizing vertebral fractures in some DISH patients. However, these studies did not assess the impact on the posterior component of the fracture, and the type of fracture (2-column or unstable 3-column) remains unclear. For unstable 3-column fractures (AO classification types B and C), additional posterior spinal fusion should be considered to enhance stability and reduce the risk of complications.

Cement-augmented percutaneous PS instrumentation also emerges as an attractive option for treating vertebral fractures in patients with DISH, offering rapid and effective fracture stabilization. The use of cement augmentation for screws in the vertebral body has been demonstrated to increase pullout loads in osteoporotic bone, surpassing normal bone pullout values [10]. Previous studies have highlighted the efficacy of percutaneous augmented instrumentation, such as cement-augmented PS instrumentation, in patients with ankylotic spine disease [15]. The successful union of fractures without losing reduction in these patients underscores the attractiveness of this surgical approach, particularly in challenging cases.

The combined application of vertebral cement augmentation and cement-augmented PS instrumentation provides robust fixation within shorter segments. When anterior support is insufficient, relying on posterior fixation alone may result in loss of reduction, hardware failure, and increased kyphosis [16]. Cadaveric studies have demonstrated that cement vertebral cement augmentation reduces pedicle screw bending moments by 59% in flexion and by 38% in extension, reducing stress on posterior instrumentation [17]. Researchers from various institutions have reported their short-term outcomes related to the use of short-segment posterior instrumentation combined with vertebral cement augmentation for managing unstable thoracolumbar burst fractures [18,19]. Cho et al. compared patients who received short-segment posterior instrumentation combined with VP to those who had only short-segment posterior instrumentation. The VP group had no instrumentation failures and maintained anterior vertebral body height while correcting kyphotic deformity, unlike the only short-segment posterior instrumentation group with a 22% failure rate [18]. Rex AW et al. presented a retrospective case series on short-segment posterior instrumentation combined with BKP in unstable thoracolumbar burst fractures (AO type B or C injuries and A3), showing excellent maintenance of reduction and low instrument failure rates [19]. Similarly, in DISH-related vertebral fractures characterized by low bone mineral density and instability, the use of VBS for anterior column reconstruction is advantageous in achieving short-segment posterior fixation.

## Conclusions

The combined application of VBS and cement-augmented PS instrumentation provides an effective and minimally invasive solution for vertebral fractures in patients with DISH. These methods contribute to the rapid stabilization of fractures, reduced postoperative complications, and improved patient outcomes.
